# Weak Handgrip at Index Admission for Acute Exacerbation of COPD Predicts All-Cause 30-Day Readmission

**DOI:** 10.3389/fmed.2021.611989

**Published:** 2021-04-07

**Authors:** Leah J. Witt, W. Alexandra Spacht, Kyle A. Carey, Vineet M. Arora, Steven R. White, Megan Huisingh-Scheetz, Valerie G. Press

**Affiliations:** ^1^Divisions of Geriatrics and Pulmonary, Critical Care, Allergy and Sleep Medicine, University of California, San Francisco, San Francisco, CA, United States; ^2^Brigham and Women's Hospital, Boston, MA, United States; ^3^Department of Medicine, University of Chicago, Chicago, IL, United States

**Keywords:** chronic obstructive pulmonary disease, patient readmission, frailty, hand strength, grip strength

## Abstract

**Rationale:** Identifying patients hospitalized for acute exacerbations of COPD (AECOPD) who are at high risk for readmission is challenging. Traditional markers of disease severity such as pulmonary function have limited utility in predicting readmission. Handgrip strength, a component of the physical frailty phenotype, may be a simple tool to help predict readmission.

**Objective(s):** To investigate if handgrip strength, a component of the physical frailty phenotype and surrogate for weakness, is a predictive biomarker of COPD readmission.

**Methods:** This was a prospective, observational study of patients admitted to the inpatient general medicine unit at the University of Chicago Medicine, US. This study evaluated age, sex, ethnicity, degree of obstructive lung disease by spirometry (FEV_1_ percent predicted), and physical frailty phenotype (components include handgrip strength and walk speed). The primary outcome was all-cause hospital readmission within 30 days of discharge.

**Results:** Of 381 eligible patients with AECOPD, 70 participants agreed to consent to participate in this study. Twelve participants (17%) were readmitted within 30 days of discharge. Weak grip at index hospitalization, defined as grip strength lower than previously established cut-points for sex and body mass index (BMI), was predictive of readmission (OR 11.2, 95% CI 1.3, 93.2, *p* = 0.03). Degree of airway obstruction (FEV_1_ percent predicted) did not predict readmission (OR 1.0, 95% CI 0.95, 1.1, *p* = 0.7). No non-frail patients were readmitted.

**Conclusions:** At a single academic center weak grip strength was associated with increased 30-day readmission. Future studies should investigate whether geriatric measures can help risk-stratify patients for likelihood of readmission after admission for AECOPD.

## Introduction

Chronic obstructive pulmonary disease (COPD) is the fourth leading cause of re-hospitalizations in the US, and over 20% of patients hospitalized for acute exacerbations of COPD (AECOPD) are readmitted within 30 days of discharge (1). Lowering 30-day readmission rates is a specific target of the Centers for Medicare and Medicaid Services, in part because re-hospitalization is expensive—the cost of all-cause re-hospitalization was estimated to be 17.4 billion in 2004 (2). On October 1, 2012, penalties began to be imposed on hospitals with high rates of unplanned readmission in six condition or procedure groups, including COPD (3). COPD is common among Medicare beneficiaries–12% of beneficiaries age 65 years and older have COPD (2); therefore reducing AECOPD readmission rates is an important goal for health systems.

The first step toward readmission reduction is identifying patients at risk of readmission. A number of retrospective studies have found that non-modifiable factors, such as severity of disease at the time of index admission, age, or socioeconomic factors like insurance type, can predict readmission among adults with COPD (4, 5). Several potentially modifiable risk factors have also been identified, such as low baseline physical activity (6) and physical frailty (7), both of which are extra-pulmonary attributes. Unfortunately, a previous meta-analysis of readmission reduction programs found no consistent benefit in readmission reduction interventions (8), though most risk reduction programs did not target modifiable extra-pulmonary risks.

Geriatric assessments, such as physical frailty evaluations and other functional assessments, are attracting interest in COPD readmission risk stratification because these extra-pulmonary factors impact all-cause readmission and mortality (9–13). Physical frailty is a geriatric syndrome of multisystem dysregulation leading to impaired physiologic and psychologic resilience. It is manifest clinically by reduced physiologic function, reduced endurance, and decreased strength (14). In the general population, frailty predominantly affects older adults and confers increased risk of hospitalizations, readmissions, and death (15, 16). Data from the National Health and Nutrition Evaluation Survey found a frailty prevalence of almost 60% in people with COPD (17). In a separate study of community-dwelling people with concomitant COPD and frailty, mortality was three-times that of non-frail patients with or without COPD. Further, frailty better predicted increased mortality better than FEV_1_ (18).

Given the high prevalence of frailty among people with COPD and the potentially intervenable nature of this syndrome, frailty may be an important extra-pulmonary risk factor that identifies patients at high risk for hospitalizations and readmissions. A 2017 study of 103 patients hospitalized for AECOPD found that frailty, measured by the Reported Edmonton Frail Scale, predicted 90-day readmission (7). Further, previous work has demonstrated that frailty is modifiable, and can improve following behavioral interventions, physical therapy or pulmonary rehabilitation (6, 19, 20). Improvements in frailty may improve disability and quality of life, as has been demonstrated in patients following lung transplantation for cystic fibrosis (21).

Handgrip strength, a component of the classic physical frailty phenotype (15), has been demonstrated in many settings to be associated with outcomes such as disability and hospital length of stay (22, 23). Handgrip weakness indicates dynapenia, which means loss of strength, and is a component of sarcopenia. Sarcopenia may be found alone or as part of the frailty syndrome (24–28). Handgrip strength is obtained via a simple bedside measurement of isometric grip strength using a commercially available handheld dynamometer. Previous work has demonstrated that low handgrip strength is predictive of the number and severity of COPD exacerbations in non-hospitalized adults, COPD mortality, and poor inhaler technique in older adults (29–31). While an alternate upper-extremity strength measure predicted all-cause COPD readmissions in a small pilot study (32), handgrip strength at index hospitalization for AECOPD has not been evaluated as a biomarker of readmission risk. The objective of this sub-study of a larger study on frailty was to test the hypothesis that handgrip strength, a feasible and simple objective assessment, could predict all-cause 30-day readmission in patients admitted for AECOPD.

## Materials and Methods

### Study Design

We conducted a prospective observational study of patients admitted to the inpatient general medicine unit at the University of Chicago Medicine, US. All participants provided written informed consent and the study was approved by the University of Chicago Institutional Review Board (14–848). This manuscript's reported findings are a subanalysis of a larger study on the interaction between frailty and COPD readmission risk.

### Study Participants

**Figure 1:**
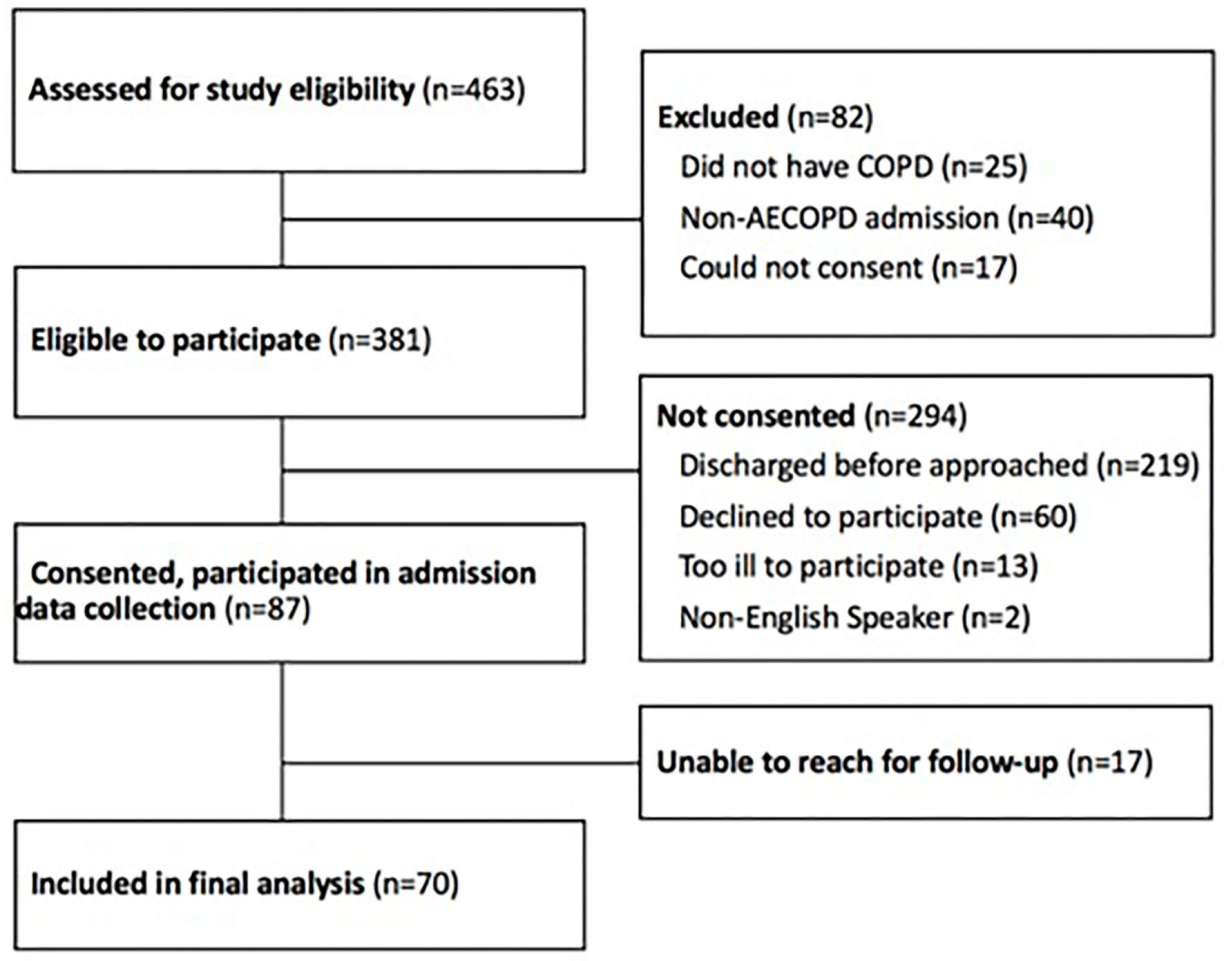
Study participants. COPD, chronic obstructive lung disease; AECOPD, acute exacerbation of chronic obstructive lung disease.

From July 2016 to January 2019, research staff screened the electronic health record (Epic Systems Corporation, Verona, WI, “EHR”) on weekdays (Monday-Friday) for patients admitted to the general medicine floor for AECOPD. The structure of care at the University of Chicago is as follows: patients with AECOPD are typically admitted from the emergency room, and cared for in the intensive care unit (which follows a closed intensive care model, in which critically ill patients are cared for by critical care physicians), or on the general medicine floor (where patients are cared for by general medicine physicians). Patients who met all inclusion criteria and met none of the exclusion criteria were considered eligible to participate (see Figure 1). Inclusion criteria included admission to a general medicine service for AECOPD and age over 18 years. Exclusion criteria were current admission to the intensive care unit and inability to give informed consent using the teachback method. Patients admitted to the ICU could become eligible for inclusion during their hospitalization upon transfer to the general medicine unit. Patients who provided written informed consent were enrolled. Patients were enrolled as they were identified but assessments did not occur at a standardized timepoint in their AECOPD hospitalization.

### Data Collection

Participant information such as comorbidities and length of stay were derived from medical records (see Supplementary Table 1). Forced Vital Capacity (FVC) and Forced Expiratory Volume in 1 s (FEV_1_) were obtained via bedside spirometry (KoKo PFT System, version 4.3, nSpire Health Inc.) by a research coordinator trained in accurate spirometry collection (33). In the larger frailty study, participants underwent two assessments for frailty and function: physical frailty phenotype (15) and the Short Physical Performance Battery (SPPB) (34). This study reports only the physical frailty phenotype results. The physical frailty phenotype is a composite score of five domains (see Supplementary Table 1): 1. handgrip strength, assessed at beside using a handheld dynamometer (Jamar Technologies Plus+, Sammons Preston, Bolingbrook, IL), and measured using the average of three isometric grip attempts (kilograms) using the dominant hand, one point for “weak grip” is assigned if the subject's grip strength is below previously published cut-points for the lowest 20th percentile for sex and BMI (15); 2. Usual gait speed, measured as the average time to complete three, 15-foot walks at usual pace, one point for “slow gait” is assigned if the subject's gait speed is below previously published cut-points for the lowest 20th percentile for sex and height subgroups (15); 3. Level of exhaustion, as determined by answers to two questions from the Center for Epidemiologic Students Depression Scale, one point for “exhaustion” is assigned if the subject answers either exhaustion question affirmatively as “a moderate amount of the time” or “most of the time”; 4. Physical activity, assessed using the six-Item Minnesota Leisure Time Physical Activity Questionnaire, one point for low physical activity is assigned for a kilocalorie expenditure <383 kcal/week for men or <270 kcal/week for women (35); and 5. Weight loss, one point for weight loss is assigned if the subject lost ≥5% of his or her body weight or 10 pounds unintentionally in the year prior to presentation (36, 37). The frailty components are binary, and one point is assigned to designate frailty in each domain. An individual is assessed to be “frail” if 3 or more domains are positive.

The primary outcome was all-cause hospital readmission within 30 days of discharge from the index AECOPD admission. Readmission data were obtained from medical record review and corroboration with follow-up phone calls if necessary.

### Statistical Analyses

Descriptive statistics were utilized to summarize the participants' characteristics. We identified associations between categorical variables using Chi-squared tests or Fisher's exact tests. Differences between groups were assessed using Student's *t-*tests or Mann-Whitney U tests for continuous variables.

Univariable logistic regression was used to assess the relationship between handgrip strength and all-cause readmission at 30 days. Univariable logistic regression was also used to assess the outcome variable of all-cause readmission at 30 days with the explanatory variables of the individual frailty components and FEV_1_ percent predicted. The models were not adjusted for age or sex, because these demographic features are already used to determine “normal” values for grip strength, gait speed, and FEV_1_ percent predicted. Data analysis and graphical analysis was conducted using STATA V.15.1 (College Station, TX). A two-tailed *p-*value of 0.05 was considered statistically significant.

## Results

Of 463 patients screened, 381 were eligible to participate based on our inclusion and exclusion criteria, and 87 consented for participation in the study (Figure 1). Of 87 participants who consented to participate in the study, 17 were unable to be reached for follow-up. The 70 remaining participants completed the study and were included in the data analysis (Figure 1).

The majority of participants were African American (91%, *n* = 64) and female (56%, *n* = 39) with a median age of 63.5 years (Quartile 1: 58.1 years; Quartile 3: 71.3 years) (Table 1). Of the participants who completed the physical frailty measures (*n* = 55), 67% were frail (*n* = 37). Of the participants who completed the grip strength maneuvers (*n* = 63), 55% had weak grip strength (*n* = 37) (Table 2).

**Table 1:** Baseline characteristics of total sample.

**Baseline characteristics**	**Total population (*n =* 70)**	**No readmission (*n =* 58)**	**Readmission (*n =* 12)**	***p-*value**
Age (years)	63.5 (58.1, 71.3)	64.3 (58.3, 71.4)	60.2 (56.2, 66.7)	0.23
Female, n (%)	39 (56%)	34 (59%)	5 (42%)	0.28
African American, n (%)	64 (91%)	52 (90%)	12 (100%)	0.58
Current smoker, n (%)	29 (41%)	24 (41%)	5 (42%)	0.99
Length of stay (days)	4.3 (2.8, 6.2)	4.3 (2.5, 6)	5 (3.3, 7.7)	0.3
Charlson comorbidity index	2 (1,3)	2 (1,3)	2 (2, 3.5)	0.21

*Data expressed as median (25th, 75th centiles)*.

**Table 2:** Univariable logistic regression predicting all-cause readmission at 30 days.

**Characteristics**	**Overall**	**No readmission**	**Readmission**	***p-*value**
Physical frailty phenotype				
Frail^a^^*^	37 (67%)	29 (62%)	8 (100%)	**0.04**
Slow walk^b^	53 (84%)	44 (83%)	9 (90%)	1.00
Weak grip^c^	36 (55%)	26 (47%)	10 (91%)	**0.009**
Weight loss^d^	17 (29%)	14 (29%)	3 (33%)	1.00
Low physical activity^e^	29 (45%)	23 (43%)	6 (55%)	0.47
Self-reported exhaustion^f^	53 (78%)	45 (80%)	8 (67%)	0.44
Spirometry				
FEV_1_ (percent predicted)^g^	32.8 (27.5, 46.3)	32.8 (27.5, 46.3)	36.3 (26.3, 57.5)	0.80

*FEV_1_ = forced expiratory volume in 1 s*.

* *frail = physical frailty score of 3 or greater*.

*Missing data*:

a *frailty score n = 15 (21%)*.

b *slow walk n = 7 (10%)*.

c *weak grip n = 4 (6%)*.

d *weight loss n = 12 (17%)*.

e *low physical n = 5 (7%)*.

f *exhaustion n = 2 (3%)*.

g *FEV_1_ % predicted n = 48 (69%). The bold values indicate significant results that are less than a p of 0.05*.

### Readmissions

Of the 70 enrolled participants, 12 (17%) were readmitted for any cause within 30 days of discharge. There were no differences in baseline characteristics among those participants who were readmitted vs. not readmitted. For instance, the average length of initial hospitalization was similar between the participants who were not readmitted and who were readmitted within 30 days (4.3 vs. 5 days, *p* = 0.3) (Table 1).

Grip strength was obtained in 66 of 70 enrolled patients, and weak grip strength was found among 91% (*n* = 10) of participants who were readmitted (*n* = 11) compared to 47% (*n* = 26) of those participants who were not readmitted (*n* = 56). For the primary outcome, weak grip during index admission predicted all-cause readmission at 30 days (OR 11.2, 95% CI 1.3, 93.2, *p* = 0.03).

None of the other individual physical frailty components were correlated with readmission in unadjusted models (Table 2). Readmission was not predicted by slow walk as measured by the 15-foot walk time assessment in an unadjusted model (OR 1.8, 95% CI 0.2, 16.4, *p* = 0.58). Readmission was not predicted by degree of airway obstruction (FEV1 percent predicted) (OR 1.0, 95% CI 0.95, 1.1, *p* = 0.7).

Of the 70 enrolled participants, 55 (78%) completed all of the Fried Frailty measures; 47 were not readmitted and 8 were readmitted. All readmitted participants were frail (*n* = 8, 100%) (*p* = 0.04). Of those not readmitted (*n* = 47), 29 participants (61%) were frail.

## Discussion

In this prospective single center study of patients hospitalized with AECOPD, weak grip strength at time of index admission predicted increased all-cause 30-day readmission. Severity of obstructive lung disease, as assessed by FEV_1_ percent predicted, was not associated with readmission. Our findings suggest that weak grip strength measured during an index admission for AECOPD may be a useful measure to identify admitted patients who are at increased risk of readmission within 30 days. This is important because many of the current tools for readmission prediction have been developed for use post-discharge, limiting the ability to act early to avoid readmissions. This simple assessment can be obtained during admission and used to triage resources to directly impact discharge planning and reduce 30-day readmissions. We also found that no non-frail participants were readmitted.

Our results have two potential implications. First, handgrip strength could be used clinically to risk-stratify patients admitted with AECOPD, as performing this assessment is simple and has more feasibility than multicomponent frailty assessments. In this study, for example, we had significant missing data in the frailty assessments due to challenges obtaining these physical measures, as one-fifth of participants did not complete all of the frailty assessments; this is in contrast to missing data from only 5% of participants for the grip strength measure. Second, use of handgrip strength to risk-stratify patients may allow for more individualized discharge planning and implementation of targeted limited resource interventions such as geriatric evaluation, outreach calls, disease education and pulmonary rehabilitation. Interventions that might modify frailty may be particularly useful in this regard.

Unfortunately most previously described readmission risk scores are underutilized for many reasons, including that some are complicated with difficult to ascertain clinical data or are validated using post-discharge data and are not validated for use during hospitalization (38–41). We hypothesize that grip strength would allow for important resources to be triaged to patients at risk during and immediately after discharge—not down the road. Identifying a just-in-time simple assessment tool to predict readmission is the goal of health systems and insurers.

Health systems aim to identify those at highest risk for readmission and implement targeted interventions that might impact readmission with the dual goals of improving patient care and garnering health care cost-savings (1, 42). Few studies have demonstrated successful interventions to reduce readmission rate after admission for AECOPD. Successful interventions include pulmonary rehabilitation, use of a discharge coordinator and interventions to teach correct inhaler use (43, 44). Unfortunately, many other interventions have had mixed success (38, 42), and others, like a comprehensive care management program and transitional care/long-term self-management support, have unexpectedly found harm (45, 46). This may be at least partially due to the fact that many readmission risk factors either are not modifiable, such as person-specific characteristics (e.g., age, insurance type) or disease severity such as low FEV_1_, or are only determined well after discharge when the patient is no longer available for immediate intervention (4, 5).

In contrast, dynapenia, for which handgrip strength is a surrogate, may be modifiable with targeted therapy interventions such as adherence to pulmonary rehabilitation, an underutilized multidisciplinary educational and exercise program for people with chronic lung disease that improves quality of life and activities of daily living, increases exercise tolerance, and reduces exacerbations (20, 47). Further, recent work demonstrates that pulmonary rehabilitation, initiated within 3 months of hospital discharge, lowers risk of mortality at 1 year among Medicare beneficiaries (48). Unfortunately, despite the strong evidence-base, pulmonary rehabilitation is not widely available in rural or resource-limited settings (49), and adherence to rehabilitation programs may be limited by factors such as patient willingness, transportation, and other social issues. Recent evidence supports more accessible web-based pulmonary rehabilitation tools to increase access for people with COPD, even those with low health efficacy (50). Home-based programs are feasible and have been demonstrated to lead to improvements in walk distance and breathlessness symptoms (51). Addressing this health disparity is critical to achieving the best care for people with COPD and perhaps for reducing readmissions for those at highest risk.

Slow gait has been demonstrated to be predictive of readmission in a 2015 study of 213 patients hospitalized for AECOPD (9). We were unable to replicate this finding in our study; this may be due to our small sample size. We also did not find an association between degree of airflow obstruction and readmission. One potential explanation for this finding is that most of our cohort had severe airflow obstruction (GOLD class 3 or 4), and few subjects had an FEV_1_ > 50% predicted. While some studies have shown that low FEV_1_ is associated with readmission, degree of airflow obstruction in general has been of limited use in identifying patients at risk for exacerbation or burdened by symptoms that impact quality of life (52). Indeed, the GOLD classification system has evolved beyond staging by FEV_1_ alone and categorizes disease severity by number of exacerbations in the prior year and degree of breathlessness (53). Additionally, prognostic calculators such as the BODE or ADO indices use degree of obstruction as only one of several inputs for mortality prediction (54, 55).

We have identified limitations to our study. Our study site was a single academic hospital, and our conclusions may not apply to non-academic hospital settings. Our sample included more African-American, women and young participants than are typically included in COPD research studies. Further research is needed to understand if findings in these understudied populations are generalizable. We believe that representation of these patients is a potential study strength. African-American populations are typically understudied, and culturally tailored interventions have been found to be effective in other chronic disease studies (56, 57). The prevalence of COPD is rising among women (58), and several of our team's previous research studies have found a higher proportion of women enrolled, which may be reflective of our study population (59, 60). Our study population was also younger than expected, which again may be reflective of the demographics of our single study site.

Due to our recruitment strategy, the frailty assessments were conducted at variable time points throughout the admission, as weekend admissions could not be evaluated until the weekday. Additionally some assessments were obtained after transfer to a general medicine floor from the intensive care unit. It is possible that those who were in the hospital for 72 h or the intensive care unit prior to undergoing a frailty assessment had experienced muscle loss due to their hospital stay. It is noteworthy that we found a significant relationship between grip strength and readmission despite this flexible enrollment. However, replication of this study with standardized timing of the frailty assessment would be helpful. Further, our response rate for enrollment was low and about 20% of consented participants were discharged before completing all of the study assessments, which may have led to a sampling bias. Because our response sample size was small, we may have been unable to detect true differences in individual component measures such as walk time and spirometry. With respect to grip strength, we did not have an objective measure of sarcopenia, such as cross-sectional area of muscle mass by CT scanning, and could not rule out that handgrip strength was confounded by conditions such as arthritis. Finally, this was a sub-analysis of a larger study of frailty markers, which is not powered to date to compare the utility of grip strength to a full frailty assessment. Larger, multi-center studies are needed to confirm our findings, and future studies should include community-based hospitals as well to determine applicability of these findings across settings.

Most readmissions following index admission for AECOPD are not due to COPD, and half are not respiratory-related (61). This period of vulnerability is referred to as post-hospital syndrome, a phenomenon of decreased function and independence following hospitalization (62). Therefore, identifying extra-pulmonary factors of global vulnerability are critical for improving care for patients with COPD; integration of geriatric and palliative care principles into COPD care would help to achieve this aim (63). Handgrip strength is a surrogate measure of overall muscle strength and conditioning, and we hypothesize that interventions that increase strength may lead to fewer AECOPD events, improved daily function, reduced hospitalizations/readmissions and prevention of post-hospital syndrome. These hypotheses require additional study.

## Data Availability Statement

The summary data supporting the conclusions of this article will be made available by the authors, upon request.

## Ethics Statement

The studies involving human participants were reviewed and approved by University of Chicago Biological Sciences Division Institutional Review Board (Protocol #14-0848). The patients/participants provided their written informed consent to participate in this study.

## Author Contributions

VP, VA, SW, and MH-S contributed substantially to the conception and design of this work. LW and AS contributed to the acquisition of the data. KC heavily contributed to the analysis of these data. KC, VP, LW, AS, and MH-S contributed to the interpretation of the data for this work. LW, AS, and VP contributed to the drafting the work. VA, SW, MH-S, and KC revised the manuscript critically for important intellectual content and accuracy. All authors gave final approval of the version to be published, gave agreement to be accountable for all aspects of the work, including the integrity of this work as a whole, from inception to published article.

## Conflict of Interest

VP has consulted for Humana, Vizient, and Roundglass. The remaining authors declare that the research was conducted in the absence of any commercial or financial relationships that could be construed as a potential conflict of interest.
