# Supplementary material for: Weak Handgrip at Index Admission for Acute Exacerbation of COPD Predicts All-Cause 30-Day Readmission
Source: Front Med (Lausanne). 2021 Apr 7;8:611989. doi: 10.3389/fmed.2021.611989 (PMC8058414; doi:10.3389/fmed.2021.611989)
Supplement: Supplementary file 1 [file Data_Sheet_1.DOCX]

**Supplement**

**Table 1**. Fried Frailty Criteria

| **Domain** | **Test** |
| --- | --- |
| **Unintentional Weight Loss** | Self-reported  Point assigned if unintentional weight loss ≥5% in previous year |
| **Level of Exhaustion** | Center for Epidemiologic Studies Depression (CESD) scale questions: “How often in the last week did you feel this way?”  0 = rarely or none of the time (<1 day)  1 = some or a little of the time (1-2 days)  2 = a moderate amount of the time (3-4 days)  3 = most of the time   1. I felt that everything I did was an effort 2. I could not get going   Point assigned if either question response is 2 or 3. |
| **Physical Activity** | Kilocalorie expenditure assessed via the Six-Item Minnesota Leisure Time Physical Activity Questionnaire  Point assigned if:  Men: <383 kcal/week  Women: <270 kcal/week |
| **Grip strength** | Hand grip strength (kilograms), measured by Jamar Technologies Plus+ hand-held dynamometer in dominant hand. Three measurements taken, reported grip strength is average of 3 measures  Point assigned if grip strength is in the lowest 20^th^ percentile (stratified by sex, BMI)   \|  \| BMI \| Pressure (kg) \| \| --- \| --- \| --- \| \|  \| *Men* \|  \| \|  \| ≤24 \| ≤29 \| \|  \| 24.1-26 \| ≤30 \| \|  \| 26.1-28 \| ≤31 \| \|  \| >28 \| ≤32 \| \|  \| *Women* \|  \| \|  \| ≤23 \| ≤17 \| \|  \| 23.1-26 \| ≤17.3 \| \|  \| 26.1-29 \| ≤18 \| \|  \| >29 \| ≤21 \| |
| **Walking speed** | Walking time in seconds (usual pace) over 15 feet  Point assigned if gait speed is in the lowest 20% (stratified by sex, height)   \|  \| Height (cm) \| Time (s) \| \| --- \| --- \| --- \| \|  \| *Men* \|  \| \|  \| ≤173 cm \| ≥7 \| \|  \| >173 cm \| ≥6 \| \|  \| *Women* \|  \| \|  \| ≤159 \| ≥7 \| \|  \| >159 \| ≥6 \| \|  \|  \|  \| |

**Frail:** ≥3 criteria present; **Pre-Frail:** 1 or 2 criteria present; **Non-Frail:** 0 criteria present

**Spirometry**

Spirometry were collected using the Koko spirometer device. Patient demographic data (sex, age, height) was entered in the Koko software to obtain patient predicted spirometry values. Collected measures were absolute and percent predicted forced expiratory volume in 1 second (FEV_1_) and forced vital capacity (FVC) as well as FEV_1_/FVC. Patients were instructed to repeat efforts until at least 3 acceptable and at least two reproducible respiratory efforts were obtained. Reproducible efforts were defined by a difference of less than 0.15 L between the largest two FVC measurements and between the two largest FEV_1_ measurements.

**References**

1. Fried LP, Tangen CM, Walston J, Newman AB, Hirsch C, Gottdiener J, et al. Frailty in older adults: evidence for a phenotype. J Gerontol A Biol Sci Med Sci. 2001;56(3):M146-56. Epub 2001/03/17. PubMed PMID: 11253156.

2. Eckel SP, Bandeen-Roche K, Chaves PH, Fried LP, Louis TA. Surrogate screening models for the low physical activity criterion of frailty. Aging Clin Exp Res. 2011;23(3):209-16. Epub 2011/10/14. doi: 8029 [pii]. PubMed PMID: 21993168; PubMed Central PMCID: PMC3351838.
